# Thrive or survive: prokaryotic life in hypersaline soils

**DOI:** 10.1186/s40793-023-00475-z

**Published:** 2023-03-13

**Authors:** Blanca Vera-Gargallo, Marcela Hernández, Marc G. Dumont, Antonio Ventosa

**Affiliations:** 1 Department of Microbiology and Parasitology, Faculty of Pharmacy, University of Sevilla, 41012 Sevilla, Spain; 2 School of Biological Sciences, University of Southampton, Southampton, SO17 1BJ UK; 3 School of Biological Sciences, Norwich Research Park, University of East Anglia, Norwich, NR4 7TJ UK

**Keywords:** Hypersaline environments, Saline soil, Stable isotope probing, Prokaryotic communities, Amplicon sequencing

## Abstract

**Background:**

Soil services are central to life on the planet, with microorganisms as their main drivers. Thus, the evaluation of soil quality requires an understanding of the principles and factors governing microbial dynamics within it. High salt content is a constraint for life affecting more than 900 million hectares of land, a number predicted to rise at an alarming rate due to changing climate. Nevertheless, little is known about how microbial life unfolds in these habitats*.* In this study, DNA stable-isotope probing (DNA-SIP) with ^18^O-water was used to determine for the first time the taxa able to grow in hypersaline soil samples (EC_e_ = 97.02 dS/m). We further evaluated the role of light on prokaryotes growth in this habitat.

**Results:**

We detected growth of both archaea and bacteria, with taxon-specific growth patterns providing insights into the drivers of success in saline soils. Phylotypes related to extreme halophiles, including haloarchaea and *Salinibacter*, which share an energetically efficient mechanism for salt adaptation (*salt-in* strategy), dominated the active community. Bacteria related to moderately halophilic and halotolerant taxa, such as *Staphylococcus*, *Aliifodinibius*, *Bradymonadales* or *Chitinophagales* also grew during the incubations, but they incorporated less heavy isotope. Light did not stimulate prokaryotic photosynthesis but instead restricted the growth of most bacteria and reduced the diversity of archaea that grew.

**Conclusions:**

The results of this study suggest that life in saline soils is energetically expensive and that soil heterogeneity and traits such as exopolysaccharide production or predation may support growth in hypersaline soils. The contribution of phototrophy to supporting the heterotrophic community in saline soils remains unclear. This study paves the way toward a more comprehensive understanding of the functioning of these environments, which is fundamental to their management. Furthermore, it illustrates the potential of further research in saline soils to deepen our understanding of the effect of salinity on microbial communities.

**Supplementary Information:**

The online version contains supplementary material available at 10.1186/s40793-023-00475-z.

## Introduction

It is estimated that salt-affected soils comprise more than 900 million hectares globally [52] and are most common in arid and semi-arid regions. Due to changing climate conditions, salinization is expected to rise continuously, with estimates that up to 50% of arable land could be drought and salt-affected by 2050 [61]. In these soils, the physical, chemical, and biological effects of salinity, together with other common concurrent factors such as relatively high temperature, low moisture, and high solar irradiation, limit plant and microbial community development. Soils are generally considered saline if they have an electrical conductivity of the saturation soil extract (EC_e_) above 4 dS/m; however, growth of sensitive crops may already be affected in soils with EC_e_ of more than 2 dS/m and only a few tolerant crops can yield satisfactorily above 16 dS/m [56]. Soils with conductivity values above this range can be considered hypersaline. High soluble salt contents impair plant development, resulting in vegetation patches and, eventually, barren land. Without vegetation, carbon inputs into the soil are greatly reduced [51], resulting in systems solely hospitable to halotolerant or halophilic microorganisms. Despite the increasing extent of saline soils, and the economic and environmental relevance of this conversion, surprisingly little is known about how microbial life unfolds in these habitats.

Previous studies on the microbial component of hypersaline soils consist of evaluations of community diversity and composition, and their relationship to physicochemical factors at several sites around the world [10, 11, 17, 29, 30, 35, 36, 58, 59, 64, 67], with few studies addressing community-wide mechanistic aspects of life in high salinity soils [12, 44, 45]. This has resulted in a poor understanding of the functioning of these systems, which is aggravated by the fact that, given the different constraints that aquatic and terrestrial habitats pose for microorganisms [4, 7], it is uncertain the degree to which the principles of halophilism and halophilic communities derived from widely studied hypersaline waters hold true in soils. In this context, incremental research on microbial life in saline soils has the potential to reveal new insights and assess the validity of extrapolations from aquatic habitats. Biodiversity profiling of saline or hypersaline soils indicates that, regardless of the life-limiting conditions of saline soils, members of archaea and bacteria from several phyla are present. Many studies have shown that halophiles belonging to the *Euryarchaeaota*, *Pseudomonadota* or *Bacteroidota* (including some *Bacteroidota* recently reclassified as *Balneolota* and *Rhodothermota*) dominate saline soils, but representatives of other taxa with diverse metabolic capabilities and susceptibilities to salt (such as *Bacillota*, *Actinomycetota*, *Cyanobacteriota*, *Chloroflexota*, *Deinococcota*, *Gemmatimonadota,* and *Planctomycetota*) are also ubiquitous in sites around the world [10, 11, 17, 29, 30, 35, 36, 58, 59, 64]. Abiotic factors such as water content, pH and soil organic carbon have been identified to play a key role in prokaryotic diversity and community structure in these soils [58, 67]. While we are now beginning to appreciate the diversity and structure of prokaryotic communities in saline soils, an understanding of their functioning is lacking. Microbial respiration and growth have been detected in naturally saline soils of up to an EC_e_ of 52 dS/m [44], however, the identity of the microorganisms involved in those activities has not been determined. Given the uncertainty about the size and nature of microbial seedbanks [22, 37, 54], it is unclear to what extent the microbial representatives detected in saline soils are active and growing at any specific moment, which traits are essential for activity in these polyextreme habitats, or what energy sources may support their activity.

Sunlight is a prevalent factor in saline soil ecosystems that could influence microbial dynamics in diverse ways. Through photosynthesis, light can be harnessed by photoautotrophs to produce new carbon compounds, which can support heterotrophic growth. Photoheterotrophs can also obtain energy for metabolic processes from light, but sunlight can also have negative effects on microorganisms via light-produced reactive oxygen species (ROS) that have deleterious effects on biomolecules. Indeed, surveys of saline soils have detected bacteria with photosynthetic metabolism (*Cyanobacteriota*, *Pseudomonadota*, *Chloroflexota*, *Gemmatimonadota* and *Bacillota*) and microorganisms with rhodopsin-based photoheterotrophic potential (*Euryarchaeota*, *Pseudomonadota*, *Bacteroidota*, *Balneolota*, *Rhodothermota*, *Cyanobacteriota*, *Bacillota*, *Actinomycetota*, *Deinococcota*, *Chloroflexota* and *Planctomycetota*). Haloarchaea, which possess a suite of adaptations that confer resistance to irradiation [18, 25, 53], constitute a large proportion of the community in these soils. With this variety of light-responsive mechanisms within these communities, light could be a key driving factor for microbial activity with consequences for energy and carbon flow.

DNA stable-isotope probing with ^18^O-enriched water (H_2_^18^O-DNA-SIP) can identify growing microorganisms in an environmental sample [1, 47]. Since water is a universal substrate for all organisms, actively growing microorganisms in a sample incubated with H_2_^18^O incorporate ^18^O into newly synthesized DNA, which can then be separated from the DNA of non-growing organisms in a density gradient. This method also allows to study the impact of environmental factors on microbial growth. It has been successfully applied to investigate soil microbial communities in diverse ecosystems [49], including extreme habitats such as McMurdo Dry Valleys in Antarctica [50].

In this study we employed H_2_^18^O-DNA-SIP coupled to high-throughput sequencing to assess prokaryotic growth in a hypersaline soil from the Odiel Saltmarshes, identifying the active taxa and physiological traits that might underpin their activity. We also evaluated the effect of light on the growing community, which provided further insights into the role of light as either a driving or inhibitory factor on microbial growth.

## Results

The soils sampled had an EC_1:5_ of 17.96 ± 5.75 dS/m (mean ± SD) (n = 3), pH of 5.5 ± 0.12 (n = 3) and gravimetric water content (WC) of 0.14 ± 0.01 g water/g dry soil (n = 6). This electrical conductivity value corresponds to approximately 97.02 dS/m in a saturated paste extract [19]. Water content was reduced to 0.08 ± 0.03 g water/g dry soil (n = 3) prior to the incubations. Previous studies showed that dissolved organic carbon (DOC) content in these soils ranged from 680.3 to 2703.2 mg/kg [58].

**Fig. 1:**
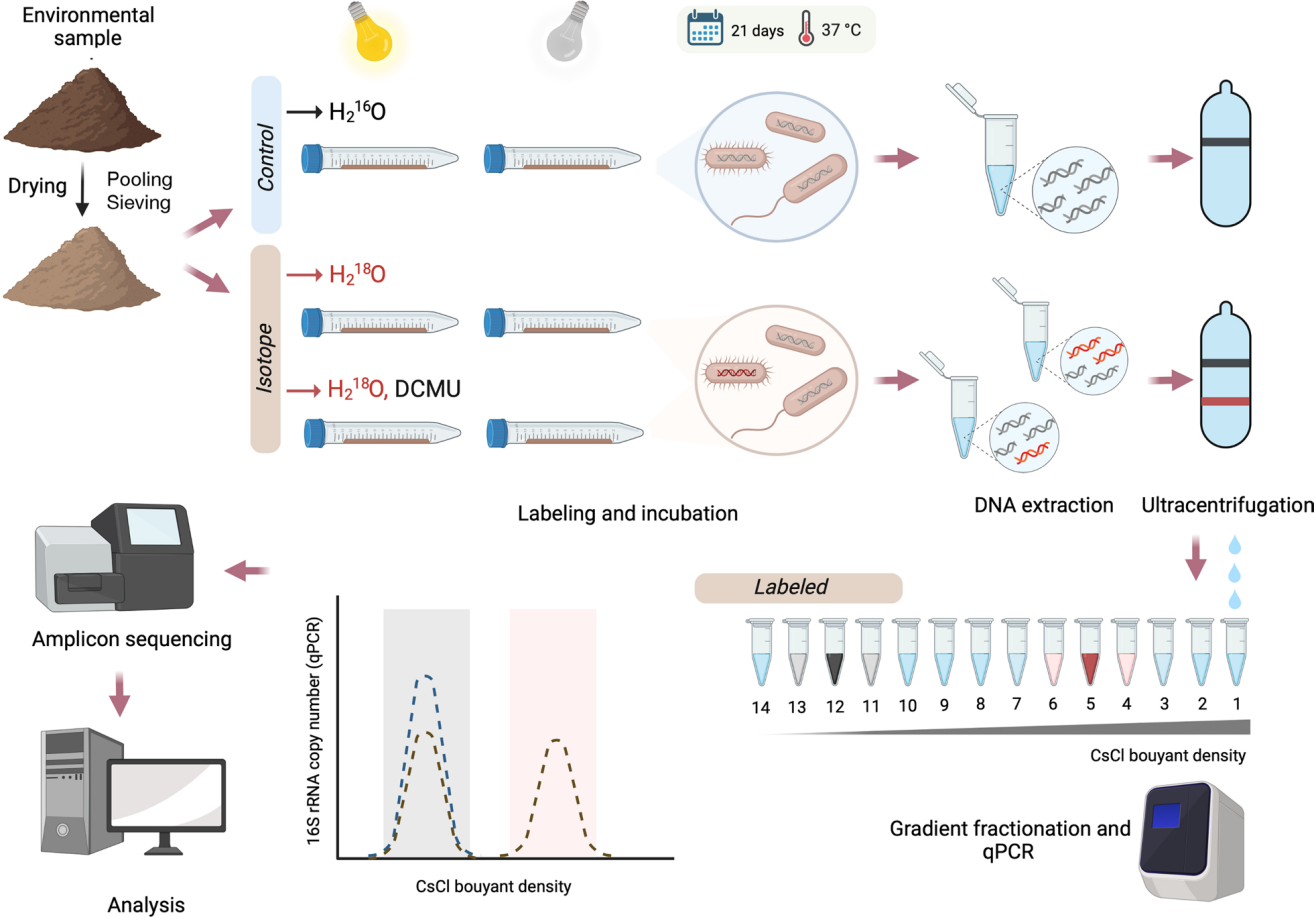
General scheme of the methodology used. First, soil was dried to approximately 50% water-holding capacity. Then, soil subsamples were subjected to each of the four treatments (Light; Dark; Light + 3-(3,4-dichlorophenyl)-1,1-dimethylurea, DCMU; Dark + DCMU) in triplicate. Sterile deionized water was used for the controls. Tubes were placed in an incubator at 37 °C for 21 days in either Light or Dark conditions. After DNA extraction, the DNA-SIP procedure was performed as described by Neufeld et al*.* [54], with slight modifications (see methods section). Archaeal and bacterial 16S rRNA genes in each of the 14 fractions obtained were quantified by real-time PCR. DNA fractions were pooled according to their buoyant densities into three groups: light DNA (L, comprising individual fractions with buoyant densities in the range 1.690–1.729 g/ml, coinciding to that of controls and therefore corresponding to unlabeled DNA), medium heavy DNA (MH, 1.730–1.749 g/ml) and heavy DNA (H, 1.750–1.780 g/ml). The binned density fractions were analyzed for 16S rRNA genes

Samples were incubated with H_2_^18^O for three weeks, which should have been sufficient time to label growing haloarchaea, which have doubling times of up to 23 days under natural conditions [34, 38]. Soil samples were incubated either in the light or dark to assess the potential role of solar illumination on microbial growth. DCMU (3-[3,4-dichlorophenyl]-1,1-dimethylurea), an inhibitor of photosystem II, was included as a treatment to differentiate between photoautotrophic and photoheterotrophic activity (Fig. 1).

**Fig. 2:**
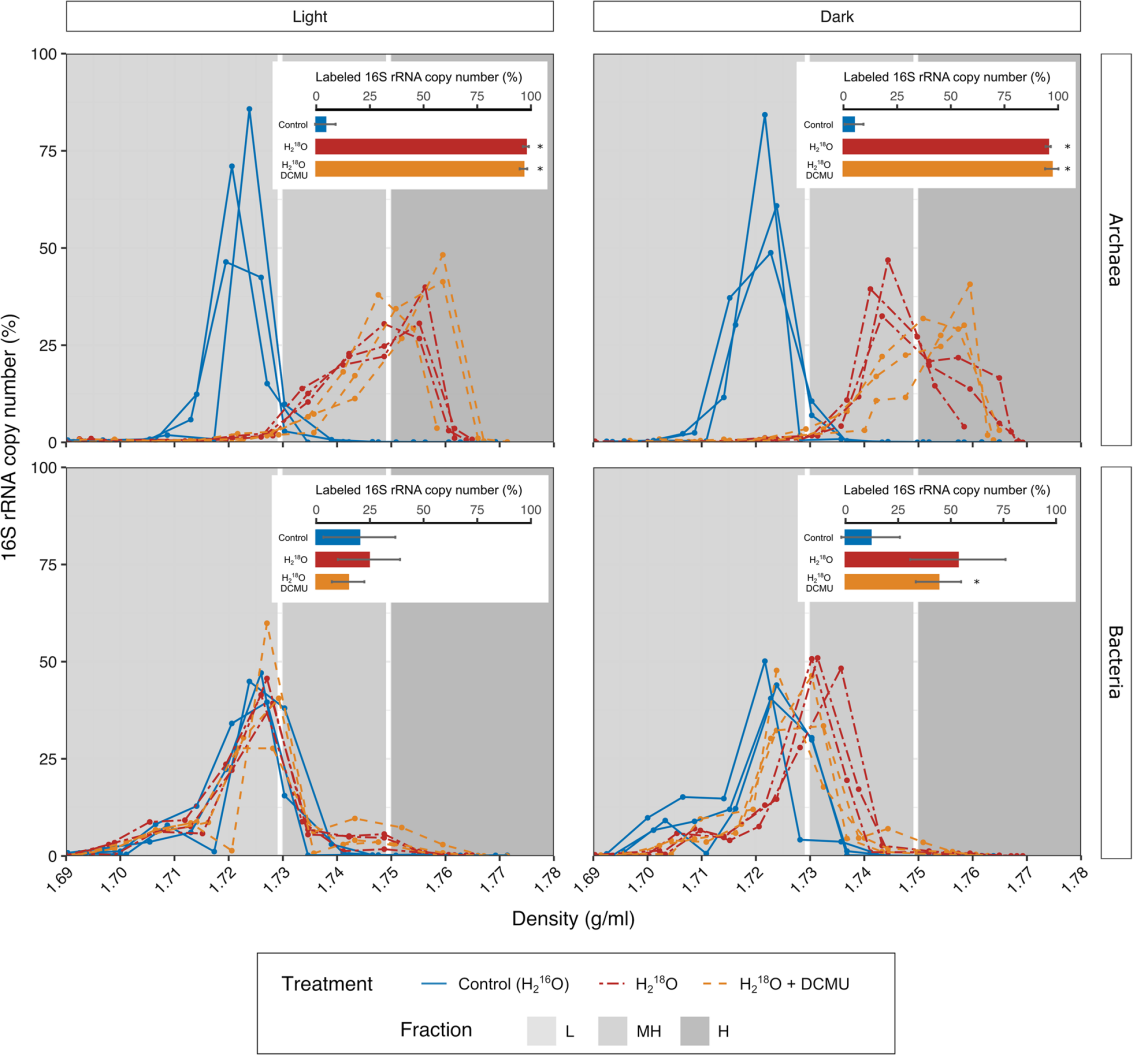
16S rRNA gene copy number of bacteria and archaea in each density fraction of gradients obtained by isopycnic centrifugation of soil DNA from different treatments. Relative values were calculated considering the total number of copies in each gradient. Each panel depicts controls and treatments (H_2_^18^O, H_2_^18^O DCMU incubated in the light or in the dark). Significant differences (*p* < 0.05, Student’s *t*-test) between controls and treatments are indicated by an (*). Bars indicate standard deviation of triplicates

Successful isotopic enrichment of DNA was achieved, as shown by the higher buoyant density of the DNA pool of ^18^O-treated samples (H_2_^18^O or H_2_^18^O + DCMU), compared to that from the H_2_^16^O-control incubations (Fig. 2). Bacterial and archaeal communities showed different patterns of labeling. In the case of Archaea, most (93–99%) of the 16S rRNA gene copies from ^18^O-incubated samples were detected in the heavy fractions (buoyant density, BD > 1.73 g/ml), regardless of the treatment employed*.* Also, the distribution of unlabeled DNA, that represents the effect of G + C content on the density of DNA, showed a narrow unimodal symmetrical distribution. In contrast, the distribution of DNA from ^18^O incubations revealed a dissimilar distribution, wider and skewed, which cannot be explained by G + C content alone. This suggest that variability in the extent of labeling of the Archaea occurred within each treatment. For Bacteria, the proportion of 16S rRNA genes appearing at buoyant densities greater than 1.73 g/ml was only apparent in the dark and this was only significantly different from control samples in the dark + DCMU incubations (*p* = 0.03, Fig. 2). The shift of DNA towards denser gradient fractions was lower for Bacteria than Archaea (Fig. 2). In H_2_^18^O-treated samples incubated in the light, just a minor fraction of bacterial 16S rRNA gene sequences appeared at higher buoyant densities than the control, with the majority showing no sign of labeling (Fig. 2).

Thirteen different phyla (*Euryarchaeota* and *Nanohaloarchaeota* in the case of Archaea, and *Actinomycetota*, *Bacteroidota*, BRC1, *Cyanobacteriota*, *Deinococcota*, *Candidatus* Dependentiae, *Bacillota*, *Gemmatimonadota*, *Planctomycetota*, *Pseudomonadota* and *Verrucomicrobiota* within Bacteria) were represented by more than 1% of the amplicons in any treatment or control (Additional file 1: Figure S1).

**Fig. 3:**
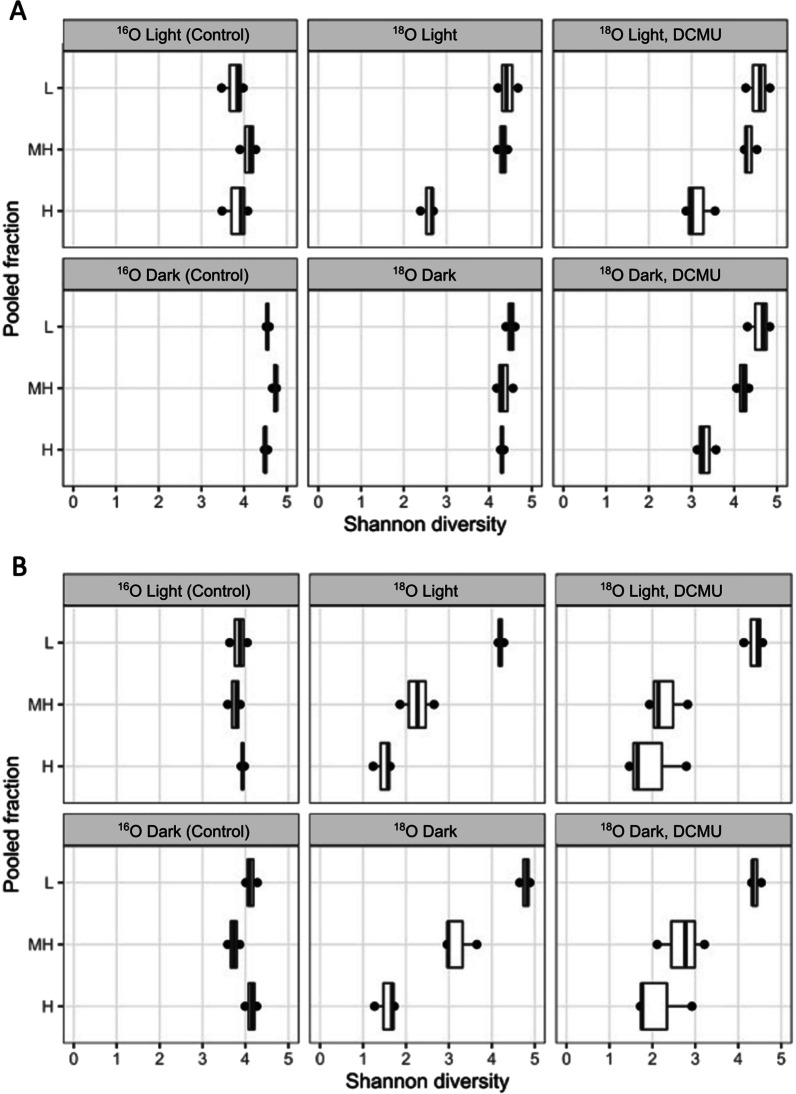
Shannon diversity index for **A** archaeal community and **B** bacterial community in each binned fraction of controls and treatments. Box and whisker plot indicate the median (horizontal line), 75th percentile (box) and maximum and minimum (points outside the box) of the distribution of diversity index for each group (*n* = 3)

Alpha diversity based on Shannon index of Amplicon Sequence Variants (ASVs) showed that archaeal diversity in the H fraction was higher in the dark incubations than in dark + DCMU, light or light + DCMU treatments (Fig. 3). The alpha diversity of the archaeal community in MH fractions was just slightly lower than in low density fractions (L). The diversity of the bacterial community decreased with density from L to H binned fractions (Fig. 3).

Phylotypes in both MH and H fractions were considered labeled if there was a significant increase in percentage of relative abundance in a dense pooled fraction of the gradient fractions of H_2_^18^O-labeled treatments compared to the corresponding pooled fraction of the unlabeled (natural abundance H_2_^16^O, control) gradient. A total of 239 phylotypes were found to be labeled in either H (78 phylotypes) or MH (146 phylotypes) pooled fractions, or both (15 phylotypes), according to the HR-SIP approach (HTSSIP), which relies on the DESeq2 algorithm to detect differences among H_2_^18^O-treatments and H_2_^16^O-controls.

**Fig. 4:**
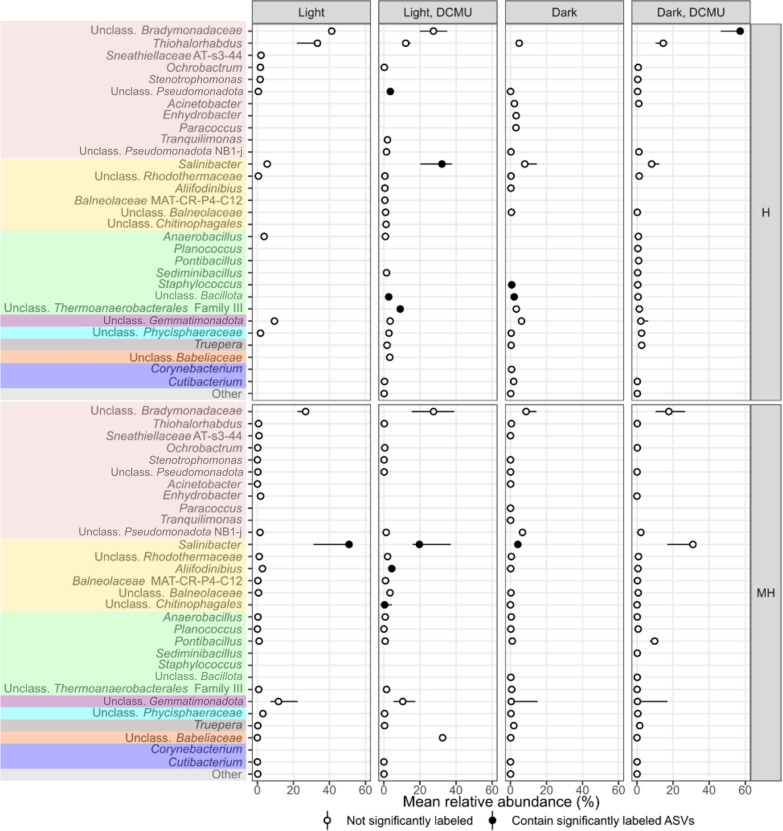
Point-range plot showing the median relative abundance (*n* = 3, points) and 50% confidence interval error bars (lines) of archaeal taxa comprising more than 1% of the reads in any treatment and heavy pooled fractions (medium heavy, MH, and heavy, H) at the genus level (or closest possible classification). Filled points indicate that at least one representative of a particular taxa was identified as significantly labeled. Colors represent different phyla –from top to bottom: *Euryarchaeota*, *Nanohaloarchaeaeota*

**Fig. 5:**
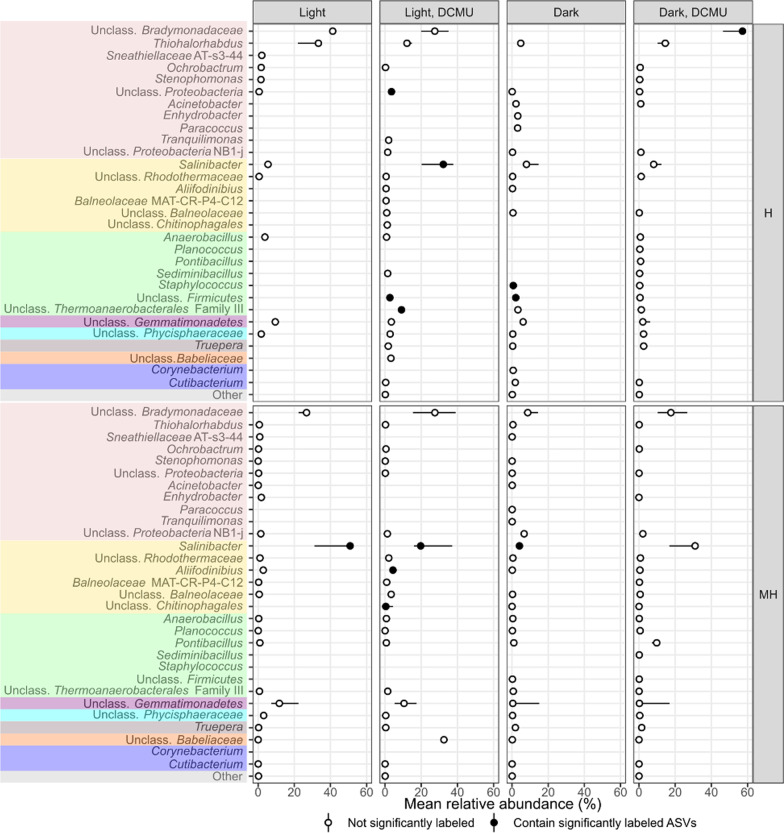
Point-range plot showing the median relative abundance (*n* = 3, points) and 50% confidence interval error bars (lines) of bacterial taxa comprising more than 1% of the reads in any treatment and heavy pooled fractions (medium heavy, MH, and heavy, H) at the genus level (or closest possible classification). Filled points indicate that at least one representative of a particular taxa was identified as significantly labeled. Colors represent different phyla –from top to bottom: *Pseudomonadota*, *Bacteroidota*, *Bacillota*, *Gemmatimonadota*, *Planctomycetota*, *Deinococcota*, *Candidatus* Dependentiae, *Actinomycetota*

Although the total number of ASVs affiliated to one or the other domain in the study was quite even (53.7% ASVs were classified as Archaea and 46.3% as Bacteria), 93.2% of labeled reads were affiliated to Archaea (224 ASVs, 21.62% of all archaeal ASVs identified in these amplicons). They spanned all known families of the class *Halobacteria* (*Euryarchaeota*), as well as some *Halobacteria* unclassified at the family level, and *Nanohaloarchaeaceae* (*Nanohaloarchaeota*). Only 15 bacterial phylotypes (1.7% of the total bacterial phylotypes detected in this study) were identified as significantly labeled by our method. These were assigned to *Salinibacter* within *Rhodothermaceae* (*Bacteroidota*), *Aliifodinibius* within *Balneolaceae* (*Bacteroidota*), unclassified *Chitinophagales* (*Bacteroidota*), unclassified *Bradymonadaceae* (*Pseudomonadota*), *Thermoanaerobacterales*-Family III (*Bacillota*), *Staphylococcus* within *Staphylococcaceae* (*Bacillota*), and unclassified members of *Bacillota* and *Pseudomonadota*. Although representatives from the domain Archaea were identified as labeled in all treatments and pooled fractions, no Bacteria-affiliated 16S rRNA gene sequence was found in the heavy fraction (H) of the light treatment (Figs. 4 and 5).

Ordination analysis of the labeled community in each sample revealed that the isotopic enrichment, as reflected in the buoyant density of the fraction where the 16S rRNA genes of labeled organisms were found (H vs MH), was a stronger determinant of differences in community composition than irradiation regime (Additional file 2: Figure S2). Nevertheless, distinct communities were labeled in the light and dark. For example, a total of 46 ASVs were identified in H fractions, but only three of these were present in both the light and the dark treatments. Of the 83 ASVs identified in MH fractions, only 12 were found in both the light and dark incubations.

Of a total of 22 labeled phylotypes of samples incubated in the light, two haloarchaeal ASVs dominated the H fractions: ASV-1267 (affiliated to the genus *Halosiccatus*) comprised between 59 to 63% of the labeled reads in these fractions, and ASV-117, related to the genus *Halorientalis*, made up 26–28% of the reads. The labeled community in the dark treatment was more diverse. Members of the phyla *Euryarchaeota* (accounting for 71.6–83.3%), *Pseudomonadota* (14.3–22.7%), *Nanohaloarchaeota* (0.2–14%) and *Bacillota* (0–1.8%) were labeled. Samples that received DCMU were dominated by a phylotype related to *Natronomonas* both in the light (76–91% in H fraction) and dark treatments (51–69% in H fraction). This phylotype was neither labeled in the light nor in the dark without DCMU. In contrast, phylotypes growing in the light and/or dark treatments were also labeled in the corresponding DCMU treatments. Furthermore, two ASVs affiliated to *Haloplanus* were identified in all H fractions, independent of the treatments applied. Known photoautotrophs were not labeled in any treatment. Importantly, we found intra-genera variability in the response to the treatments.

## Discussion

This study aimed to identify the actively growing prokaryotes in saline soils from the Odiel Saltmarshes, also dissecting the potential role of light on their growth. Although the microbial community in saline soils has been investigated previously, the activity and growth of these populations within the soil matrix had not been previously studied. We used H_2_^18^O-SIP, which allows the identification of actively replicating organisms [48] to monitor growth in situ. Both bacterial and archaeal DNA could be detected in heavy fractions after incubating the soil samples with H_2_^18^O-SIP (with or without the inhibitor of photosynthesis, DCMU) for three weeks (Fig. 2), which indicates that representatives of both domains incorporated the isotope, which is a proxy for growth. Notably, different levels of growth were identified across taxa. Archaea outperformed bacteria in terms of i) proportion of total DNA residing in gradient heavy fractions, ii) labeling degree, iii) tolerance to assayed light regimes (Fig. 2), and iv) proportion of ASVs labeled. In general, the low proportion of bacterial phylotypes labeled, together with the weak labeling of bacteria and their sensitivity to light suggested the labeled bacteria were growing closer to their physiological growth limit. Further insights about the mechanisms underlying these global labeling patterns were sought through evaluation of the growth response at finer taxonomic levels.

Of the 13 phyla that comprised more than 1% of the reads in any pooled fraction (Additional file 1: Figure S1), only *Euryarchaeota*, *Nanohaloarchaeota*, *Bacteroidota*, *Pseudomonadota* and *Bacillota* were represented in the growing community (Figs. 4 and 5). Among labeled taxa, those related to the extremely halophilic haloarchaea (*Euryarchaeota*) and *Salinibacter* (classified as *Bacteroidota* in the databased employed, but now recognized as belonging to the phylum *Rhodothermota*), dominated these soils. These two phylogenetically unrelated organisms share a unique and efficient osmoadaptation strategy that provides them with a clear potential advantage in terms of growth at high salinity. Life in hypersaline environments is energetically costly [32]. By employing KCl for osmotic adjustment of their cytoplasm (the so-called *salt-in* strategy), the maintenance requirements of *salt-in* strategists are reduced to only a fraction of the cost of the *salt-out* strategy used by other halophiles, which often depend on the synthesis of osmolytes [32, 33]. In this way, extreme halophiles could allocate a higher proportion of their energy budget into physiological functions other than osmoadaptation, including biomass production. This is argued as the reason why, as observed in hypersaline aquatic systems, these organisms can withstand higher upper salinity limits and have higher in situ growth rates than organisms relying on the *salt-out* strategy [34].

Remarkably, the predominance of extreme halophiles in these soils seems to occur at comparatively lower conductivities than in hypersaline aquatic bodies [5, 43, 55]. We hypothesize that, together with the uncertainties around the ability of current soil salinity evaluations (electrical conductivity in soil extracts) to accurately represent the actual osmotic stress faced by microorganisms in soil systems in a given moment [26], additional stressors exclusive to soils may select for energetically efficient organisms at lower measured conductivity levels. In soils, apart from the osmotic effect resulting from the amount of solutes, the matric effect (derived from the presence of solid particles and their nature) further limits water availability, hence posing an extra energetic burden for maintaining cell turgor, repairing oxidative damage, and obtaining water for cellular functions. Furthermore, microorganisms in such soils may experience a shortage of energy sources (either by scarcity or by diffusion limitation). In this context, while the concentration of salts is the predominant life-limiting factor in many of the hypersaline aquatic systems studied to date and, therefore, salinity is highly correlated with microbial community composition there, it may not be possible to predict community composition of microbial communities from saline soils exclusively from salinity levels. Overall, our findings are compatible with the relative importance of salinity as a primary life-limiting factor being higher in hypersaline aquatic systems than in soils. These results also suggest the *salt-in* mechanism provides a broad benefit in energy-demanding situations. Nevertheless, further evaluations of the correlation of EC with the salinity effectively experienced by soil microorganisms are needed to validate these hypotheses.

Together with those affiliated to haloarchaea and *Salinibacter*, some phylotypes related to moderate halophiles or halotolerant bacteria were also labeled (Figs. 4 and 5). Several mechanisms may be involved in the growth of these organisms in this study. Spatiotemporal heterogeneity of soil offers a plausible explanation. In saline soils, heterogeneity could lead to temporally and spatially isolated microhabitats with diverse physicochemical composition that may shelter organisms with a variety of salt tolerances or requirements [54, 57]. At the same time, labeled taxa possess features that may be especially suited for life in these habitats. For example, the labeling of a phylotype related to *Staphylococcus*, a genus with recognized ability to produce exopolysaccharides (EPS), points towards this mechanism being advantageous in saline soils. EPS protect against drought and salinity and serves as a carbon storage polymer, among others [13]. Additionally, the identification of growing organisms related to *Bradymonadaceae*, a recently described group that thrives through bacterivory [28], suggests that a predatory lifestyle may benefit microbes in certain situations in these soils. With predation being reduced with decreasing soil moisture [46], it may seem contradictory to propose it as advantageous in unsaturated soils. In this regard, labeled taxa could have been actively growing during the whole three-week period or, alternatively, just at a particular moment. The latter could be the case of phylotypes related to *Bradymonadaceae*, which might have had a greater opportunity to prey during rehydration of the soil. As for *Chitinophagales*, many of its described members can metabolize complex polymers such as lignin or chitin, which would provide them with selective advantage through utilization of plant or insect-derived materials that would sporadically arrive to the system. Finally, it is unclear from the limited available literature what properties could provide representatives related to *Aliifodinibius* with additional advantage over other moderate halophiles in this habitat. These, as well as other labeled unidentified taxa, may possess unrecognized strategies specifically suited for life in saline soils that await to be described.

The growing phylotypes incorporated the isotope to different extents, indicating that the described properties or combination of them may be advantageous to different degrees. Furthermore, traits associated with resource acquisition (e.g., versatility of carbon and energy sources, and motility) or the type of energy-producing metabolism also influence growth rate. Since nutrient acquisition has been identified as a phylogenetically labile trait [16], nutritional differences might contribute to explain the variability observed in the growth response of different ASVs affiliated to the same genus or of phylotypes inferred to employ the same osmoadaptation mechanism.

Differences in the main source of oxygen incorporated into DNA could also be partly responsible for variations in labeling. Organisms may incorporate oxygen from external water, metabolic water, or other oxygen-containing organic compounds [1, 21, 23]. It is difficult to assess the contribution of these factors since there is limited information regarding the main pathways of oxygen incorporation by taxa.

Distinct onset of growth after rehydration could similarly result into different degrees of labeling. There is evidence that microorganisms display a specific onset of response to either rewetting events or addition of carbon sources, that is, upon alleviation of water and/or nutrient limitation [46]. This phenomenon has been attributed to mechanisms of metabolic recovery, in the short term, or coordinated sequential utilization of substrates or feeding relationships over a longer term [1, 20, 39]. With haloarchaea being particularly resistant to desiccation [53], it would not be surprising if they resumed their growth rapidly after dry periods, providing another foundation for their observed advantage. Phylotypes in MH and H could also lead different lifestyles or be engaged in trophic relationships [49].

In this study, light limited the growth of most Bacteria (Fig. 2) and hampered the development of a diverse archaeal community (Fig. 3). This is in line with the reduction of prokaryotic diversity in response to increased luminic intensity detected in previous studies in salterns [60]. In both cases, *salt-in* strategists were favored over *salt-out* Bacteria in the light (compared to dark) or at higher luminic irradiation (*versus* low luminic irradiation). Apart from potential undetermined growth-enhancing effects of light on haloarchaea and *Salinibacter* in these soils, negative impacts of irradiation could also contribute to the configuration of different communities as a function of the light regime. The deleterious effects of light on biomolecules, mainly mediated by the generation of reactive oxygen species (ROS), require extensive and specific repair that not all organisms would equally handle. In this regard, haloarchaea have a wide variety of systems devoted to protecting and repairing ROS-induced damage to cellular structures, which have been evaluated in the context of their high tolerance to UV and IR irradiation [62]. No such detailed studies are available for halophilic bacteria, yet there is some evidence that most of them could be less resistant to oxidative stress than haloarchaea [55]. Furthermore, in the proposed framework of energetics having a key role in prokaryotic success in saline soils, organisms relying on *salt-in* strategy would also be superior to *salt-out* since they would potentially have a higher proportion of their energetic budget available to be expended in repairing light-induced damage.

Importantly, no phylotype affiliated to groups with described photosynthetic capability became labeled in any incubation, which also suggests that prokaryotic photosynthesis may not play an important role under the conditions we tested. We also considered whether eukaryotic photoautotrophs may be active. In the absence of prokaryotic photosynthesis, differences between samples incubated in the light with and without DCMU may suggest either eukaryotic photosynthetic activity or photoheterotrophic effects. DCMU treatments showed a high inter-replicate variability and, importantly, signs of an off-target effect of the inhibitor (Figs. 4 and 5), which precluded confident interpretations being drawn. The literature indicates that algae from the genus *Dunaliella* (*Chlorophyta*) supports the heterotrophic community in hypersaline systems such as saltern ponds [38], although it is apparently absent in some other hypersaline systems [2]. This genus has been detected in saline soils [3, 8], but previous metagenomic datasets from the particular site studied here showed no sequences related to *Chlorophyceae* [59]. The exact ecological role of photosynthetic groups and energy and carbon flow in saline soils should be determined in further studies and natural light settings in these environments as well as in soil crusts. In the apparent absence of fresh photosynthate production in the studied conditions, organic carbon already present in the soil samples when collected as well as cell debris originated in drying-rewetting processes may potentially constitute important energy and carbon sources.

It should be noted here that the resolution of the method employed to identify significantly labeled phylotypes within this dataset depends on the number of fractions sequenced and the extent of label incorporation of each phylotype. Furthermore, the power of statistical methods for detecting significance of enrichment decline when the number of labeled organisms is high, such as in H_2_^18^O SIP studies. With three pooled fractions sequenced (L, MH, and H) this study will have detected only the most successful taxa in the system, which was sufficient for the purpose of this investigation.

## Conclusions

Overall, this study explored the drivers of prokaryotic growth in saline soils and the influence of light, leading the way toward a mechanistic understanding of the microbial ecology and functioning of these systems. Extreme halophiles, predominantly belonging to haloarchaea, were identified as the most successful organisms indicating the importance of extremely halophilic *Euryarchaeota* in these systems. Nevertheless, a relatively large number of moderately halophilic or halotolerant bacterial species appeared to be growing close to their physiological growth limit, raising important ecological questions about the relative contribution of extreme halophiles and less adapted populations to soil functioning in these systems as well as the potential for the communities in degraded soils to recover. This study also reveals that the relative importance of salinity as a primary life-limiting factor may be higher in saline waters compared to terrestrial hypersaline habitats, challenging direct extrapolations from one system to the other.

## Materials and methods

### Sampling, physicochemical characterization, and sample preparation

Three core soil samples (4-cm depth, 10 cm apart from each other and at least 60 cm away from surrounding plants) were collected in August 2017 from a saline unvegetated patch from the Odiel Saltmarshes (GPS coordinates 37.207218, -6.965999), located in Huelva (Southwest Spain) and selected based on the availability of previous studies in this same area that described the prokaryotic diversity, spatial dynamics, and metabolic potential [58, 59]. Samples were kept cold for transportation to the laboratory. A thin 1–2 mm top layer consisting mainly of dry salt crystals was discarded, and the three soil samples were mixed while subjected to wet sieving through a 2-mm mesh at 4 °C. Water content was measured gravimetrically in an oven at fixed temperature until a constant weight was reached. The pH and electrical conductivity were determined in 1:5 (w/v) extracts. Electrical conductivity in saturated paste extract was estimated from EC_1:5_ using published relationships [19].

### H_2_^18^O-SIP experiment design and incubations

Prior to incubation the soil was dried to approximately 50% water-holding capacity by placing a 1-cm layer in a sterile box containing CaCl_2_ and leaving it to dry at 37 °C, periodically monitoring moisture by gravimetry.

Soil subsamples (2 g) were weighed into 15-ml Falcon tubes and subjected to each of the four treatments in triplicate: 30 µl of H_2_^18^O (CK Isotopes, Ibstock, UK) + (i) Light; (ii) Dark; (iii) Light + 5 µM 3-(3,4-dichlorophenyl)-1,1-dimethylurea (DCMU, Sigma-Aldrich); (iv) Dark + 5 µM DCMU. Sterile deionized water (H_2_^16^O) was used for the controls. The moistened soil was thoroughly mixed and spread along the tube wall to make a thin layer of a few millimeters thick. Tubes were placed in an incubator at 37 °C for 21 days. Light setting consisted of two 40 W LED cylindrical bulbs providing 147.5 ± 10 µmol quanta/m^2^ s of 5500 K light. Dark conditions were achieved by covering corresponding tubes with aluminum foil. The Falcon tubes were opened weekly to avoid anoxic conditions to develop.

### DNA extraction, isopycnic centrifugation and gradient fractionation

FastDNA SPIN Kit for Soil (MP Biomedicals, California, USA) was used to extract DNA from 0.5 g of each subsample at the end of the incubation period. We followed the DNA-SIP procedure described by Neufeld et al*.* [31], with slight modifications. For isopycnic centrifugation, 1 µg of DNA was loaded into a 5.1 ml Quick-Seal (Beckman Coulter) tube with gradient buffer and CsCl solution achieving a final density of 1.725 g/ml. Samples were spun on an Optima XPN 80 ultracentrifuge (Beckman) with a Beckman VTi90 rotor for 60–65 h at 47,200 rpm and 20 °C (Dumont and Hernández, 2019). The tube contents were fractionated in 14 fractions of 350-µl each by pumping water with a syringe pump at a constant rate (700 µl/min), and the density of each fraction was measured with a Reichart AR200 digital refractometer. DNA in each fraction was precipitated overnight using 2 µl of linear acrylamide (Ambion, 5 mg/ml) and 2 volumes of PEG solution (30% PEG 6000, 1.6 M NaCl).

### Quantification of 16S rRNA genes in gradient fractions

Archaeal and bacterial 16S rRNA genes in each fraction were quantified by real-time PCR using TaqMan assays on an AB StepOnePlus instrument with StepOne software v2.3 (Applied Biosystems). Archaea and bacteria were targeted separately using the primer and probe combinations Arc787F-Arc1059R-Arc915P for Archaea and BAC338F-BAC805R-BAC516P for Bacteria [66]. The probe was synthesized with 6-carboxyfluorescein (6-FAM) on the 5’ end and Black Hole Quencher 1 (BHQ1) on the 3’end (Sigma-Aldrich). Standards were prepared by PCR of cloned genes and serially (10^1^–10^6^ diluted for calibration curves in each assay. Each reaction had a final volume of 10 µl and consisted of 5 µl of TaqMan Fast Advanced Master Mix (1X (Thermofisher, 0.5 µl of primer/probe mix [18 µl of forward primer (0.9 µM, 18 µl reverse primer (0.9 µM, 5 µl of probe (0.25 µM, and 59 µl of TE buffer], 1.0 µl of DNA template and 3.5 µl of water. The same program was used for both assays and consisted of 95 °C for 5 min, followed by 35 cycles of 95 °C for 30 s and 62 °C for 60 s [66]. The coefficients of determination (*R*^2^) for the assays ranged from 0.996 to 0.99 and amplification efficiencies fell between 86 and 103%.

DNA fractions were pooled according to their buoyant densities into three groups: light DNA (L, comprising individual fractions with buoyant densities in the range 1.690–1.729 g/ml, coinciding to that of controls and therefore corresponding to unlabeled DNA), medium heavy DNA (MH, 1.730–1.749 g/ml) and heavy DNA (H, 1.750–1.780 g/ml). The binned density fractions were analyzed for specific gene targets to facilitate community characteristics calculations as described below. Pooled samples were sent to the Environmental Sequence Facility at the University of Southampton (UK) for 16S rRNA amplicon sequencing of the V4 region with universal primers 515FB and 806RB [15]. These universal primers, standard in the sequencing facility, were proposed by the Earth Microbiome Project protocol for 16S rRNA Illumina amplicon sequencing. They are known to cover bacteria as well as *Euryarchaeota* (the main archaeal representatives in these hypersaline environments) and *Thaumarchaeota*. Nevertheless, the efficiency of universal primers for detecting other Archaea has been found to differ from that of kingdom-specific primers [6], and thus, disparities may arise when comparing qPCR results to amplicon sequencing data.

### 16S rRNA amplicon analysis/bioinformatic analysis

Adaptor and primer sequences were removed from the dataset using cutadapt 1.16 [24]. Amplicon Sequence Variants (ASV) identification, removal of chimeras and taxonomic assignations with SILVA NR database version 132 [41] was carried out with DADA2 package version 1.10 [9] in R 3.6.2 for macOS [42] using functions sort, filterAndTrim (maxEE = c(2,2), truncQ = 2, rm.phix = TRUE), learnErrors, derepFastq, dada, mergePairs, removeBimeraDenovo, assignTaxonomy and addSpecies. In this version of the SILVA database nanohaloarchaea were included within *Nanoarchaeota* phylum. To avoid confusion, when the class, order and family indicated that the sequences belonged to *Nanohaloarchaeota*, the name of the phylum was corrected. Also, names of phyla have been updated according to the current phyla nomenclature. The R package phyloseq v. 1.24.0 [27] was employed for further analysis. ASVs not present in at least 3 samples were removed. This step filtered out 2019 ASVs from 40 different phyla. Collectively, these ASVs comprised less than 2% of the reads in each sample. Individually, none of them made more than 0.003% of the reads in any sample. Alpha diversity trends are not modified by this step (data not shown). This prevalence filter was employed to ensure removal of artifacts related to sample processing. Excluding ASVs that were only rarely seen among samples increased the power of subsequent statistical testing of significantly labeled taxa. Sequences were then aligned with DECIPHER v2.14 package [63] function AlignSeqs and a phylogenetic tree was built with FastTree 2.1.11 [40] for macOS (default mode for nucleotides). Identification of labeled ASVs was carried out by comparison with the same pooled fraction in the control (unlabeled) gradients as implemented in HR-SIP method within HTSSIP package v1.4.1 [65]. Non-metric multidimensional scaling (NMDS) ordination was computed based on Bray Curtis dissimilarity of squared root transformation of abundance data of labeled phyla calculated at the ASV level with function ordinate (transform = FALSE) from the phyloseq was used for plotting. Sequence data was deposited in the Sequence Read Archive (SRA) under the bioproject accession number PRJNA634977.

## Supplementary Information

**Additional file 1: Fig. S1.** Barplot of **A** archaeal and **B** bacterial phyla represented by more of 1 % of the reads in composite fractions L (comprising individual fractions with buoyant densities in the range 1.690–1.729 g/ml), MH (individual fractions with buoyant densities in the range 1.730–1.749 g/ml), and H (1.750–1.780 g/ml) of each control and treatment, which comprised the following conditions: 2 g of sample + 30 µl of H_2_^18^O + (i) Light / (ii) Dark /(iii) Light + 5 µM 3-(3,4-dichlorophenyl)-1,1-dimethylurea (DCMU) / (iv) Dark + 5 µM DCMU.

**Additional file 2: Fig. S2.** NMDS analysis showing the relationship among the growing **A**) archaeal, **B**) bacterial, **C**) overall communities in each treatment (Light / Dark / Light + 5 μM 3-(3,4-dichlorophenyl)-1,1-dimethylurea (DCMU) / Dark + 5 μM DCMU) and binned fraction (L, comprising individual fractions with buoyant densities in the range 1.690–1.729 g/ml; MH, consisting of fractions with buoyant densities in the range 1.730–1.749 g/ml and H, encompassing fractions with buoyant densities in the range 1.750–1.780 g/ml). Differences are based on Bray Curtis dissimilarity calculated at the ASV level. Colors and shapes indicate different treatments and binned fractions.

## Data Availability

The datasets generated and analyzed during the current study are available in the Sequence Read Archive (SRA) with accession numbers SRX8395748 to SRX8395801. The bioproject accession number is PRJNA634977 (https://www.ncbi.nlm.nih.gov/bioproject/?term=PRJNA634977).
