# Correction: Thrive or survive: prokaryotic life in hypersaline soils

**DOI:** 10.1186/s40793-023-00491-z

**Published:** 2023-04-20

**Authors:** Blanca Vera-Gargallo, Marcela Hernández, Marc G. Dumont, Antonio Ventosa

**Affiliations:** 1 Department of Microbiology and Parasitology, Faculty of Pharmacy, University of Sevilla, 41012 Sevilla, Spain; 2 School of Biological Sciences, University of Southampton, Southampton, SO17 1BJ UK; 3 School of Biological Sciences, Norwich Research Park, University of East Anglia, Norwich, NR4 7TJ UK

Correction: **Environmental Microbiome (2023) 18:17 **10.1186/s40793-023-00475-z

Following publication of the original article, the authors flagged that they had provided incorrect versions of figures 4 and 5. The article has since been updated with these figures and the figures may be seen in this erratum. The authors thank you for reading this erratum and apologize for any inconvenience caused.

**Fig. 4:**
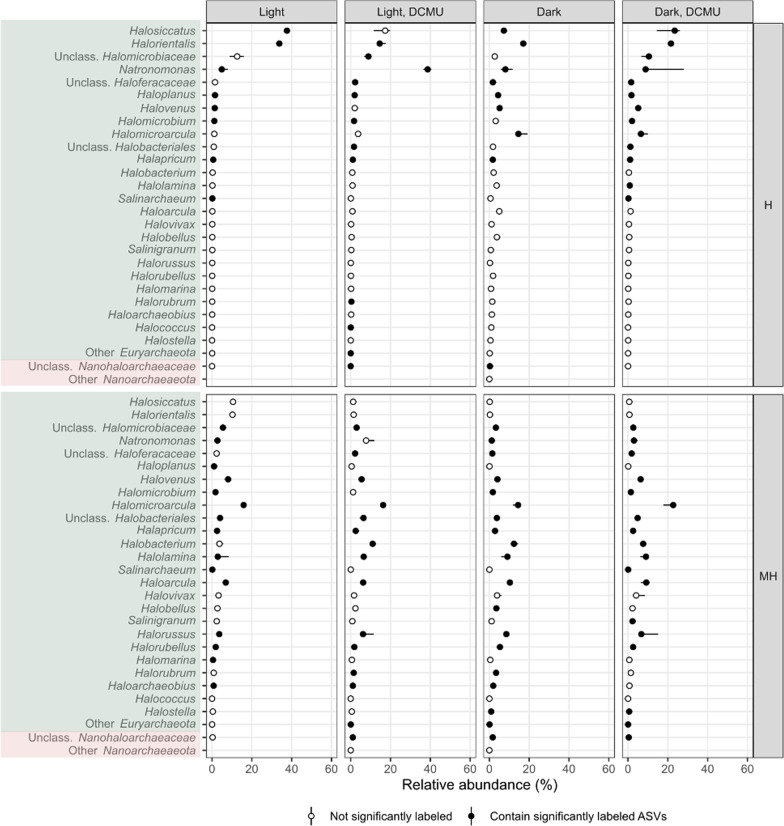
Point-range plot showing the median relative abundance (*n* = 3, points) and 50% confidence interval error bars (lines) of archaeal taxa comprising more than 1% of the reads in any treatment and heavy pooled fractions (medium heavy, MH, and heavy, H) at the genus level (or closest possible classification). Filled points indicate that at least one representative of a particular taxa was identified as significantly labeled. Colors represent different phyla –from top to bottom: *Euryarchaeota*, *Nanohaloarchaeaeota*

**Fig. 5:**
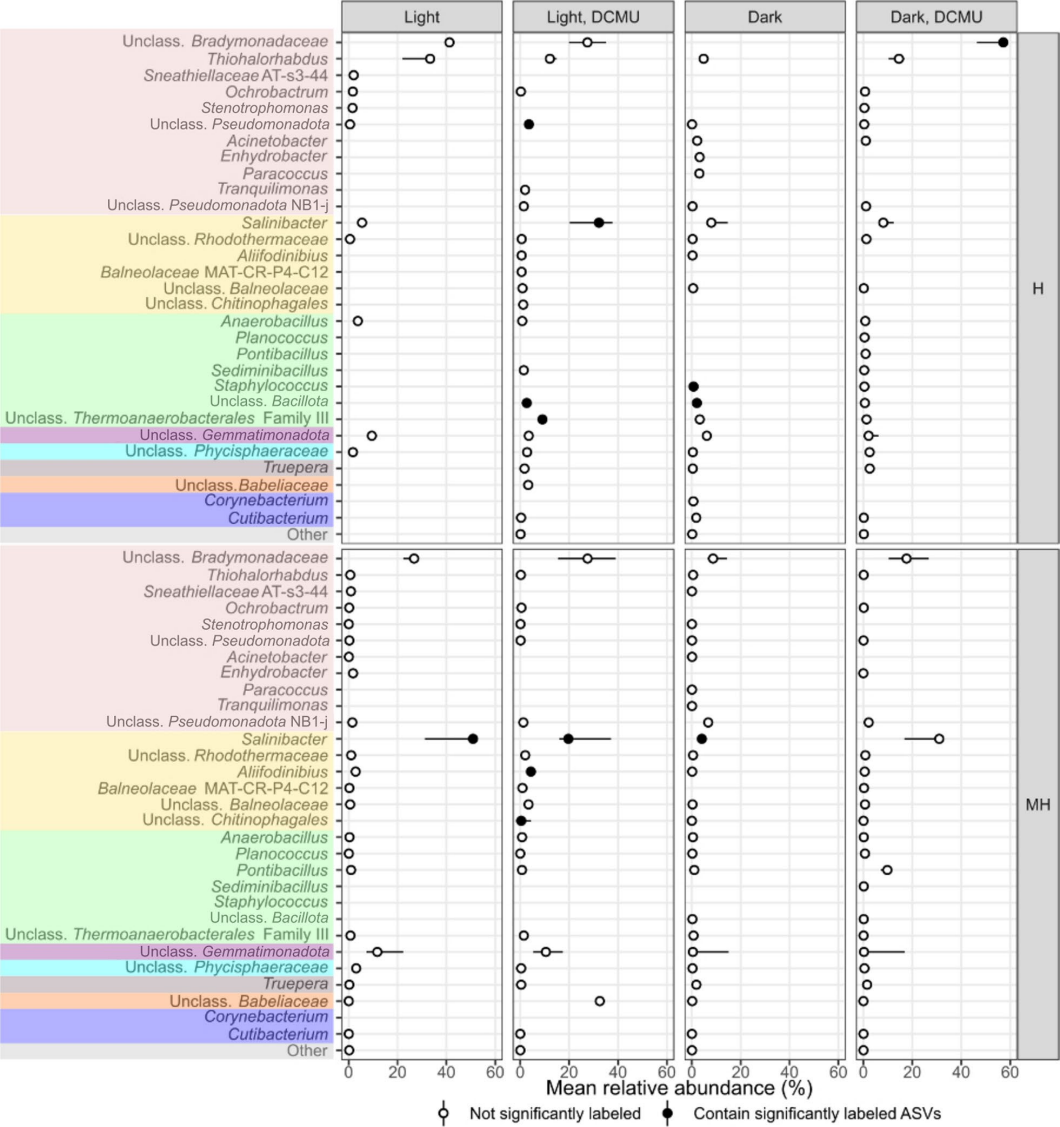
Point-range plot showing the median relative abundance (*n* = 3, points) and 50% confidence interval error bars (lines) of bacterial taxa comprising more than 1% of the reads in any treatment and heavy pooled fractions (medium heavy, MH, and heavy, H) at the genus level (or closest possible classification). Filled points indicate that at least one representative of a particular taxa was identified as significantly labeled. Colors represent different phyla –from top to bottom: *Pseudomonadota*, *Bacteroidota*, *Bacillota*, *Gemmatimonadota*, *Planctomycetota*, *Deinococcota*, *Candidatus* Dependentiae, *Actinomycetota*

