# Supplementary material for: Thrive or survive: prokaryotic life in hypersaline soils
Source: Environ Microbiome. 2023 Mar 13;18:17. doi: 10.1186/s40793-023-00475-z (PMC10012753; doi:10.1186/s40793-023-00475-z)
Supplement: Supplementary file 1 — Additional file 1: Fig. S1. Barplot of A archaeal and B bacterial phyla represented by more of 1 % of the reads in composite fractions L (comprising individual fractions with buoyant densities in the range 1.690–1.729 g/ml), MH (individual fractions with buoyant densities in the range 1.730–1.749 g/ml), and H (1.750–1.780 g/ml) of each control and treatment, which comprised the following conditions: 2 g of sample + 30 µl of H218O + (i) Light / (ii) Dark /(iii) Light + 5 µM 3-(3,4-dichlorophenyl)-1,1-dimethylurea (DCMU) / (iv) Dark + 5 µM DCMU. [file 40793_2023_475_MOESM1_ESM.pdf]

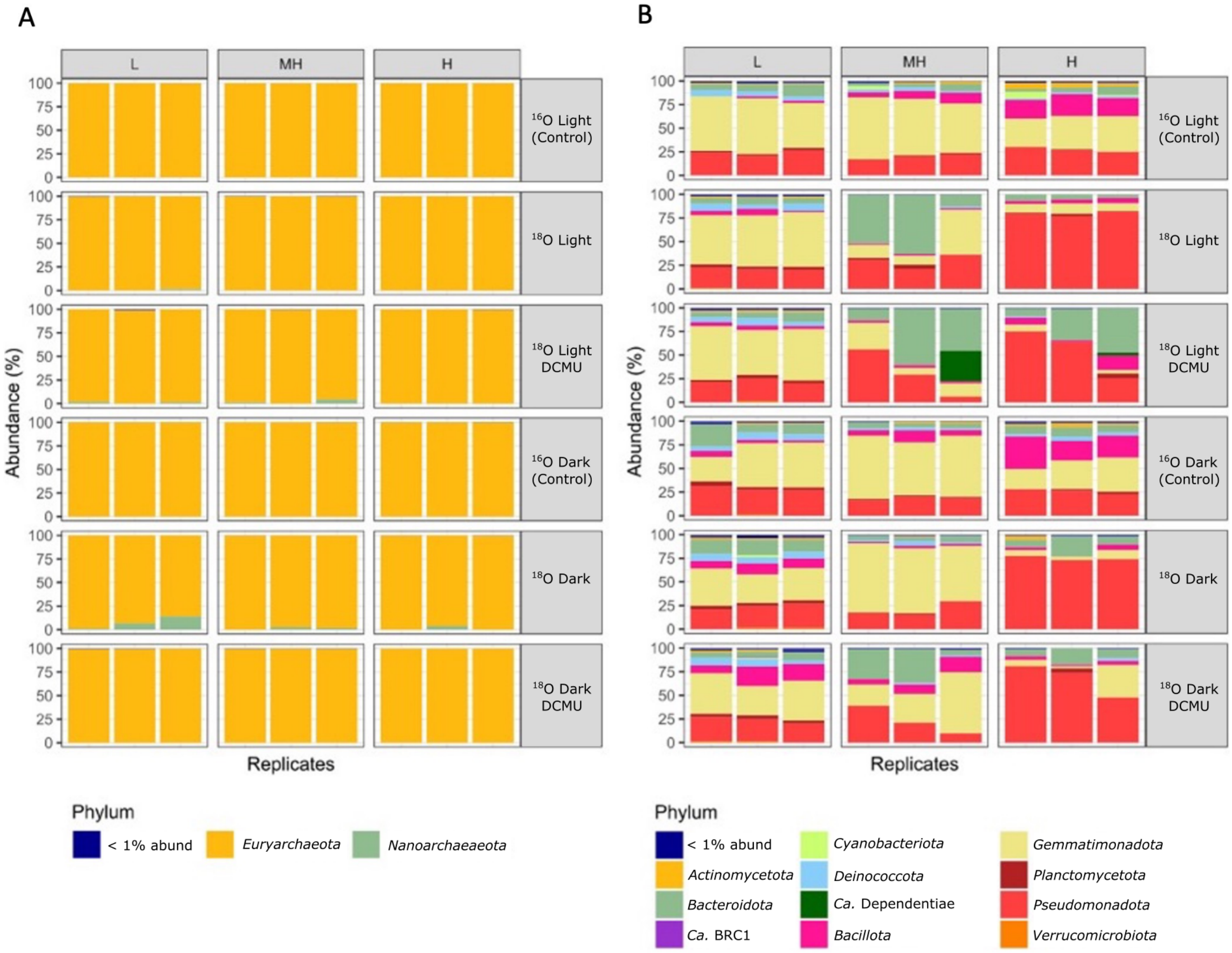

Supplementary Figure S1. Barplot of (A) archaeal and (B) bacterial phyla represented by more of 1 % of the reads in composite fractions L (comprising individual fractions with buoyant densities in the range 1.690-1.729 g/ml), MH (individual fractions with buoyant densities in the range 1.730-1.749 g/ml), and H (1.750-1.780 g/ml) of each control and treatment, which comprised the following conditions: 2 g of sample + 30 µL of $H_2{}^{18}O$ + (i) Light / (ii) Dark /(iii) Light + 5 µM 3-(3,4-dichlorophenyl)-1,1-dimethylurea (DCMU) / (iv) Dark + 5 µM DCMU.
