# Supplementary material for: Thrive or survive: prokaryotic life in hypersaline soils
Source: Environ Microbiome. 2023 Mar 13;18:17. doi: 10.1186/s40793-023-00475-z (PMC10012753; doi:10.1186/s40793-023-00475-z)
Supplement: Supplementary file 2 — Additional file 2: Fig. S2. NMDS analysis showing the relationship among the growing A) archaeal, B) bacterial, C) overall communities in each treatment (Light / Dark / Light + 5 μM 3-(3,4-dichlorophenyl)-1,1-dimethylurea (DCMU) / Dark + 5 μM DCMU) and binned fraction (L, comprising individual fractions with buoyant densities in the range 1.690–1.729 g/ml; MH, consisting of fractions with buoyant densities in the range 1.730–1.749 g/ml and H, encompassing fractions with buoyant densities in the range 1.750–1.780 g/ml). Differences are based on Bray Curtis dissimilarity calculated at the ASV level. Colors and shapes indicate different treatments and binned fractions. [file 40793_2023_475_MOESM2_ESM.pdf]

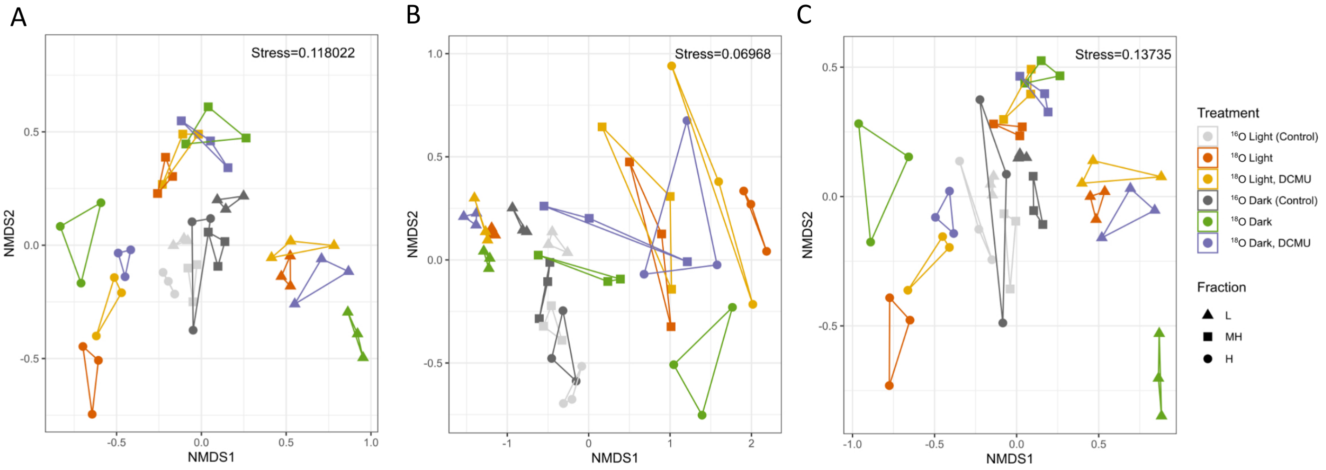

Supplementary Figure S2. NMDS analysis showing the relationship among the growing A) archaeal, B) bacterial, C) overall communities in each treatment (Light / Dark / Light + 5 µM 3-(3,4-dichlorophenyl)-1,1-dimethylurea (DCMU) / Dark + 5 µM DCMU) and binned fraction (L, comprising individual fractions with buoyant densities in the range 1.690-1.729 g/ml; MH, consisting of fractions with buoyant densities in the range 1.730-1.749 g/ml and H, encompassing fractions with buoyant densities in the range 1.750-1.780 g/ml). Differences are based on Bray Curtis dissimilarity calculated at the ASV level. Colors and shapes indicate different treatments and binned fractions.
